# Supplementary material for: Bridging non-overlapping reads illuminates high-order epistasis between distal protein sites in a GPCR
Source: Nat Commun. 2020 Feb 4;11:690. doi: 10.1038/s41467-020-14495-7 (PMC7000732; doi:10.1038/s41467-020-14495-7)
Supplement: Supplementary file 1 — Supplementary Information [file 41467_2020_14495_MOESM1_ESM.pdf]

Supplementary Information

Bridging non-overlapping reads illuminates high-order epistasis between distal protein sites in a GPCR

Yoo et al.

Supplementary Note 1: Detailed explanation of BRIDGE methodology

Below, we provide a detailed explanation of the BRIDGE methodology:

Illumina next-generation sequencing (NGS) platforms are almost exclusively used for deep mutational scanning (DMS) studies due to their high accuracy and throughput[6]. Illumina NGS platforms employ sequencing-by-synthesis (SBS) technology wherein an engineered DNA polymerase replicates a DNA strand in the presence of fluorescently-labeled dNTPs with modified 3' hydroxyl groups, which enable step-wise addition and identification of dNTPs[7]. Upon incorporation of a labeled dNTP, an optical system detects the identity of the nucleotide, then fluorophore release and 3'-modification is catalyzed allowing the next labeled dNTP to be incorporated. The resulting sequence is called a read, which can reach up to 300 bases in length using current technology. Parallelization is achieved through the use of a sequencing chip surface modified with two unique DNA adapters, which bind DNA strands carrying complementary, flanking sequences that can be appended to any strand through PCR (**Supplementary Figure 14**).

In a process referred to as bridge amplification, the distal ends of bound DNA strands approach the chip surface facilitating interaction between the complementary sequences in the template and on the surface. The DNA template is replicated from the second adapter generating a covalently-bound DNA strand. Accordingly, millions of unique DNA strands adhere to the surface and are clonally amplified through repeated rounds of bridge amplification to generate clusters comprising thousands of identical, proximal copies, significantly improving the signal-to-noise ratio[8]. Bridge amplification also enables paired-end sequencing, wherein a single DNA strand is sequenced from both ends providing additional sequence information or improving accuracy if the paired-end reads overlap (**Fig. 1b**). However, due to limitations in read length (i.e. ≤ 300 bases), paired-end reads will only overlap if the 5'-ends of each read are separated by < 600 bases. Thus, distal mutations separated by ≥ 600 bases preclude sequencing using overlapping paired-end reads.

In addition to reducing sequencing accuracy, non-overlapping paired-end reads preclude the use of available DMS software to match reads arising from the same DNA strand. Identification of all mutations arising in a single gene is crucial due to the poorly predicted effects of epistasis. We provide a straightforward method to extend DMS to protein libraries containing distal mutations by leveraging the proximity with which paired-end reads generate fluorescent signals upon incorporating nucleotides into the synthesized read (**Fig. 1b**). For a given DNA strand, the fluorescent signals emitted from each paired-end read share an (x, y) coordinate on the sequencing chip surface. As spatial coordinates are provided for each read in the output FASTQ files, we developed code to parse through the forward and reverse FASTQ read files and match reads that share an (x, y) coordinate.

Following cluster generation, fluorescent signals originating from distinct clusters may overlap on the chip surface reducing the accuracy of matching paired-end reads emerging from the same DNA strand. The occurrence of these polyclonal clusters can be reduced by optimizing clustering density, which can be controlled by the concentration of DNA loaded into the instrument flow cell. However, raw sequencing data passes through a "chastity" or "purity" filtering step in which instrument software automatically discards reads with overlapping fluorescent signals. Accordingly, the sequences contained in FASTQ files are likely to represent those originating from monoclonal clusters.

Supplementary Note 2: Application of BRIDGE to libraries containing >2 distal loci

Although our library was designed to group all mutations in two distal loci, BRIDGE is not limited by positional constraints within individual reads. The mutations may be located anywhere within the length of either read; however, these locations should be chosen taking into consideration the practical considerations noted in **Supplementary Note 3**. As an example, we illustrate below the application of BRIDGE to a protein library wherein mutations are placed in three separate loci (**Supplementary Figure 15**).

Using the Illumina NextSeq sequencer, read lengths reach up to 150 bases enabling use of a reverse read that captures mutations imparted at $L249^{6.51}$, $H250^{6.52}$, and $S281^{7.46}$, which resides on transmembrane helix 7 and has been reported to alter ligand binding affinity upon mutagenesis[9,10]. Standard protocols provided by the manufacturer can be used to prepare and sequence the NGS library. Upon obtaining forward and reverse FASTQ read files, BRIDGE can be applied to match non-overlapping paired-end reads generated from the same DNA strand.

The code utilized to match non-overlapping paired-end reads (i.e. append_reads.py) does not have to be modified as it relies solely on flow cell coordinates encoded within the FASTQ sequence identifier and is sequence-agnostic. To trim and quality-filter reads using code applied in this manuscript, the trim-qc() function can be modified as noted in the README.md file. Upon matching and appending paired-end reads, analysis of enrichment rates may be performed using available software (e.g. Enrich2), or the code described in this manuscript can be modified accordingly. Specifically, appended reads can be translated to their corresponding amino acid sequences by modifying and applying the translate_reads() function.

Supplementary Note 3: Practical considerations in applying BRIDGE.

Upon attachment to the sequencing chip surface, DNA templates undergo bridge amplification to facilitate cluster generation and paired-end sequencing (**Supplementary Figure 14**). Thus, amplification efficiency, which is generally greater for shorter templates[18], is important for successful bridge amplification and sequencing of paired-end reads. Accordingly, paired-end sequencing is often suggested for templates with length ≤ 1 kb[19]. However, different libraries may yield varying results. While we predict most templates with length ≤ 1 kb will be compatible with BRIDGE, this methodology can likely be applied to longer templates. Provided successful bridge amplification, BRIDGE can be applied to match paired-end reads produced from the ends of the DNA template (i.e. loci separated by ≤ 1 kb).

Prior to analysis of any template, it is prudent to determine optimal cluster density for the specific DNA template. As the density of clusters on the sequencing chip surface increases, the likelihood of exceeding the optimal cluster density (i.e. overclustering) also increases. Overclustering results in a reduced fluorescence signal-to-noise ratio and increases the difficulty of generating and resolving distinct clusters. Although achieving a cluster density that is less than optimal (i.e. underclustering) retains high data quality, it reduces the amount of data that is obtained from an NGS run. Optimal cluster densities for various Illumina NGS platforms are provided by the manufacturer. The concentration of library DNA leading to optimal cluster density can be determined empirically through instructions provided by the manufacturer.

Note that the efficiency of cluster generation can also be impacted by other variables including nucleotide diversity in the initial cycles. Nucleotide diversity and the efficiency of cluster generation is often increased through the addition of PhiX or 1 – 3 nucleotides in the primers used to generate the NGS library. Manufacturer instructions should be noted in optimizing cluster density.

As noted in **Supplementary Note 1**, raw sequencing data passes through a “chastity” or “purity” filtering step in which instrument software automatically discards reads with overlapping fluorescent signals. Therefore, the sequences contained in FASTQ files are likely to represent those originating from monoclonal clusters.

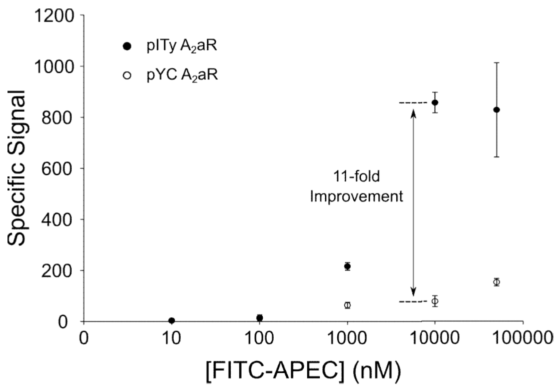

Supplementary Figure 1: Expression of *A_2aR* from a multisite integrating vector (pITy) improves specific fluorescent ligand binding signal compared to expression from a conventional centromeric backbone (pYC). A fluorescent ligand (FITC-APEC) binding assay demonstrates up to an 11-fold improvement in specific signal obtained in yeast producing A_2aR using a multisite integrating vector compared to the less mitotically stable, low-copy centromeric vector. Data represent the mean of three biological replicates, and error bars represent their standard deviation. Source Data are provided as a Source Data file.

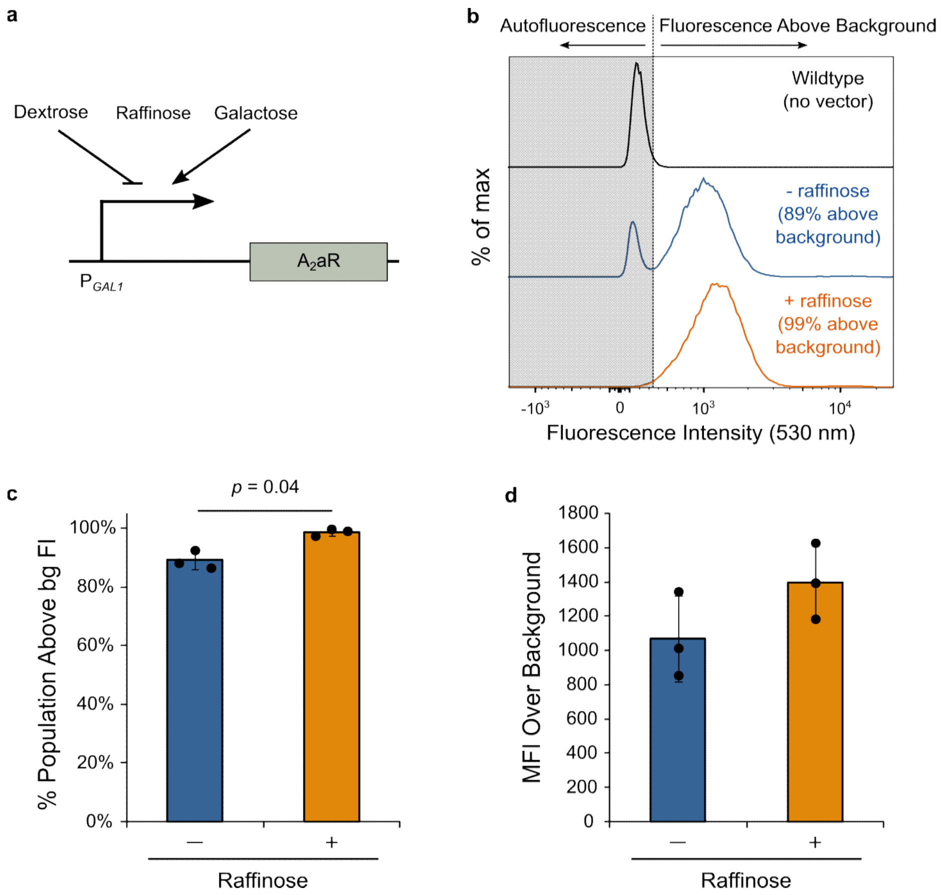

Supplementary Figure 2: Growth with raffinose prior to induction of P_{GAL1}-driven *A_2aR* expression improves phenotypic homogeneity upon incubation with fluorescent ligand. (a) Expression of *A_2aR* under the control of the P_{GAL1} promoter is repressed by dextrose, induced by galactose, and unaffected by raffinose. (b, c) Flow cytometric analysis of yeast serially cultured in dextrose followed by galactose reveals phenotypic heterogeneity, where 10% of the population exhibits fluorescence intensities comparable to autofluorescence in wildtype yeast (i.e. yeast not transformed with pITy A_2aR). In contrast, cells serially cultured in dextrose, raffinose, and raffinose combined with galactose are phenotypically homogeneous with 99% of the population exhibiting fluorescence intensities above background. (d) The addition of a raffinose growth step marginally improves mean fluorescence intensity (MFI) over background. *A_2aR* expression in this experiment was performed using a pITy A_2aR construct. In panel b, each histogram corresponds to a representative sample. Data represent the mean of three biological replicates, and error bars represent their standard deviation. Statistical significance was determined through calculation of *p* values using a paired, two-sided Student's t-test. Source Data are provided as a Source Data file.

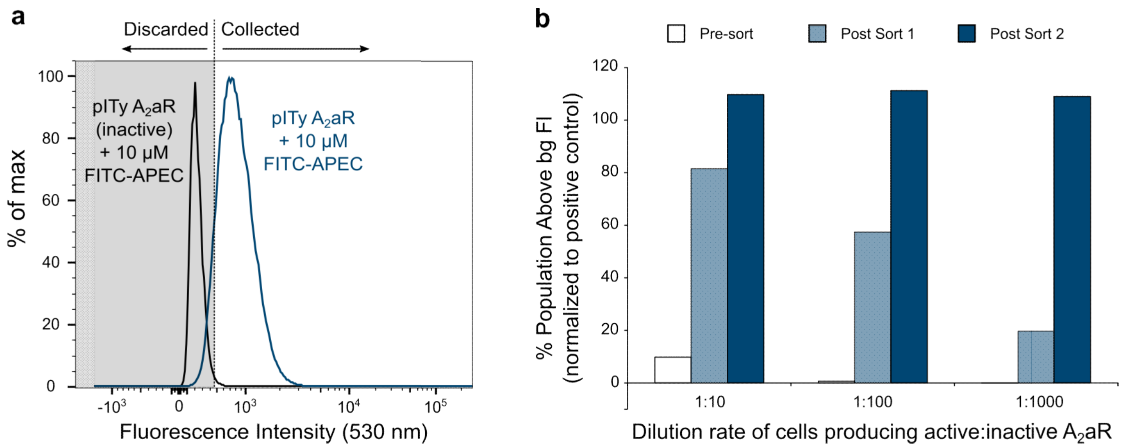

Supplementary Figure 3: Optimization of FACS parameters enables complete enrichment of yeast producing active A_2aR diluted into a pool of cells producing an inactive A_2aR variant. In order to assess the sorting stringency achieved using our GPCR expression and fluorescent ligand binding protocols, we performed a pilot enrichment experiment. Since our goal is to isolate GPCR variants based on their ligand binding properties, we screened a mixed population comprising wildtype A_2aR and an inactive A_2aR variant (C28A/C82A/C128A/C185A/C245S/C254A/C394S) that does not bind ligand. (a) Flow cytometric analysis of pure populations incubated with 10 µM FITC-APEC demonstrates the expected phenotypes and allow us to position the sort gate to isolate cells producing active A_2aR. (b) Yeast cultures producing wildtype A_2aR and the inactive variant were incubated with fluorescent ligand, mixed in varying dilution rates, and sorted using FACS. Only 2 rounds of sorting are required to fully enrich cells producing active A_2aR from a pool diluted 1:1000 with cells producing the inactive variant. In panel a, each histogram corresponds to a representative sample. In panel b, data represent the results of a single experiment with one sample for each dilution rate.

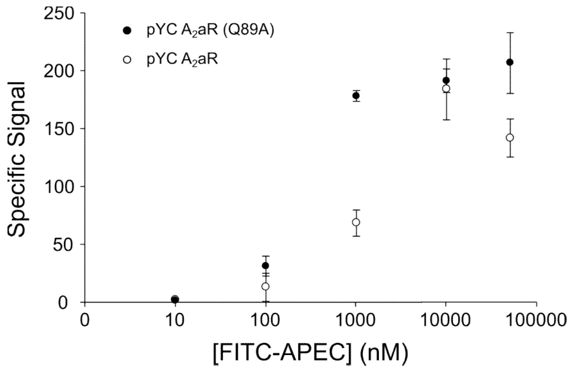

Supplementary Figure 4: Optimized GPCR expression and FACS screen discriminates between A_2aR variants based on ligand binding affinity. A saturation fluorescent ligand binding experiment was performed using yeast producing wildtype (WT) A_2aR and a variant (Q89A) reported to exhibit improved binding affinity to NECA, an adenosine receptor agonist structurally similar to APEC. Specific signal reflects the improved binding affinity anticipated in yeast producing A_2aR (Q89A) compared to cells producing the WT receptor. In this experiment, A_2aR genes were expressed from the low-copy, non-integrating pYC backbone, which is used to express the A_2aR library. Data represent the mean of three biological replicates, and error bars represent their standard deviation. Source Data are provided as a Source Data file.

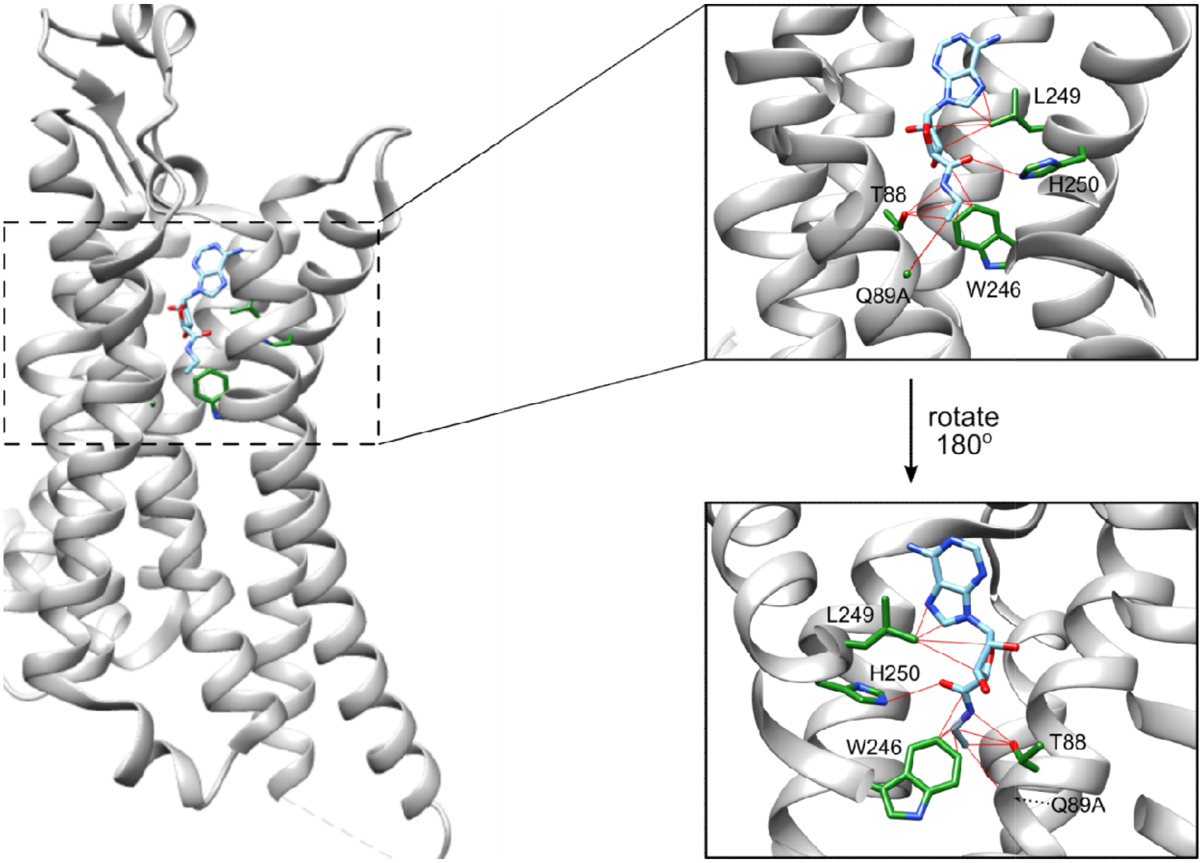

Supplementary Figure 5: Structure of the adenosine A_2a receptor bound to NECA. The crystal structure of A_2aR bound to NECA (PDB code 2YDV[8]) is shown including stick representation of side chains belonging to residues investigated in this study (i.e. T88$^{3.36}$, Q89$^{3.37}$, W246$^{6.48}$, L249$^{6.51}$, H250$^{6.52}$). The shown structure was generated using a thermostabilized variant, which contains a C-terminal truncation at residue 316 and 4 substitutions including a Q89A mutation. Red lines connect atoms between the ligand and side chains whose van der Waals radii are separated by ≤ 0.4 Å.

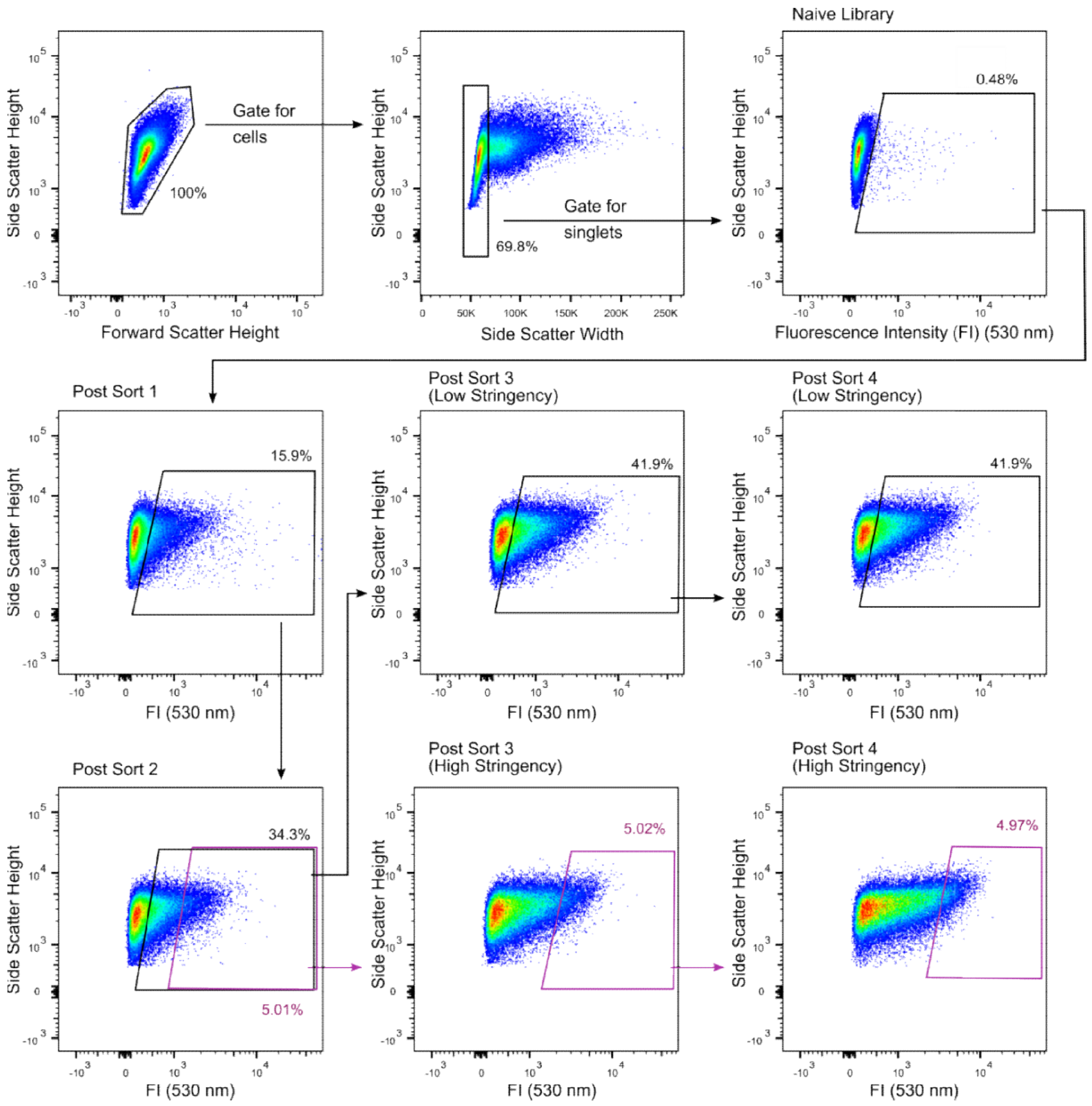

Supplementary Figure 6: Illustration of high- and low-stringency FACS sorting strategies. All library populations were initially gated to sort cells and singlets. In the first two rounds of sorting, all cells above background fluorescence intensity were collected. The background fluorescence intensity was determined for each round of sorting using an empty vector (pYC) negative control. In the third round of sorting, the Post Sort 2 population was sorted using both high- and low-stringency gating. In the high-stringency gating strategy, we collected the top 5% of cells with respect to mean fluorescence intensity. In the low-stringency gating strategy, we collected all cells displaying mean fluorescence intensity above background. In the fourth round of sorting, either high- or low-stringency gating was repeated for the respective population.

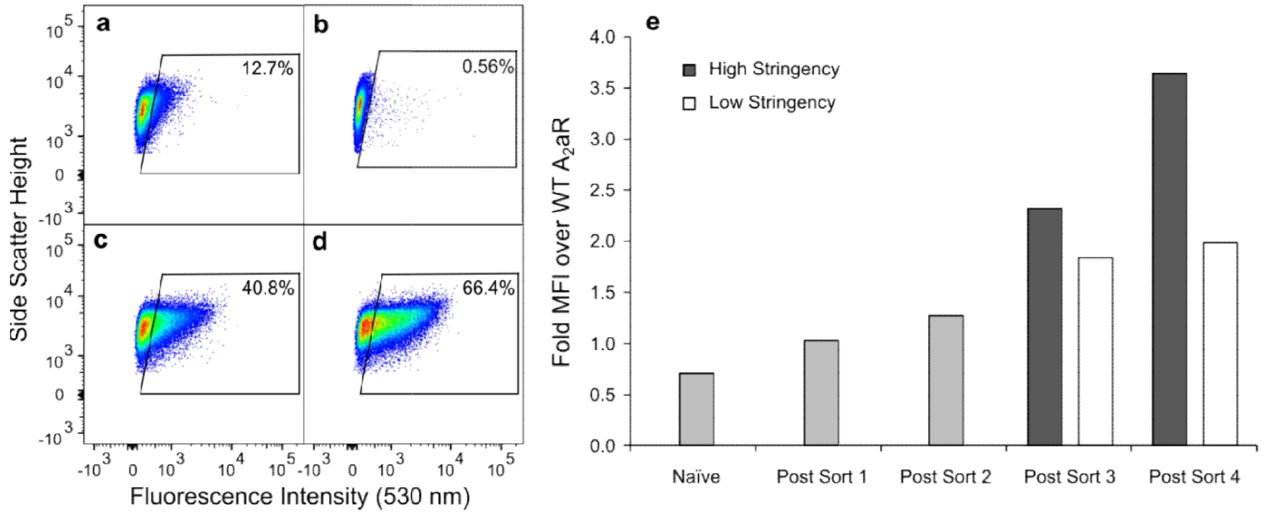

Supplementary Figure 7: Implementation of a FACS-based GPCR screen enriches A_2aR variants with highly improved ligand binding or expression phenotypes. Flow cytometric dot plots of (a) wildtype (WT) A_2aR and (b) naïve, (c) PS4 low-stringency, and (d) PS4 high-stringency libraries incubated with 10 μM FITC-APEC reveal enrichment of cells with improved ligand binding phenotypes, which are greatest in the PS4 high-stringency population. In the dot plots, red and blue represent high and low densities, respectively. (e) Cellular mean fluorescence intensity (MFI) is sequentially improved after each round of FACS suggesting enrichment of A_2aR variants with improved binding or expression phenotypes.

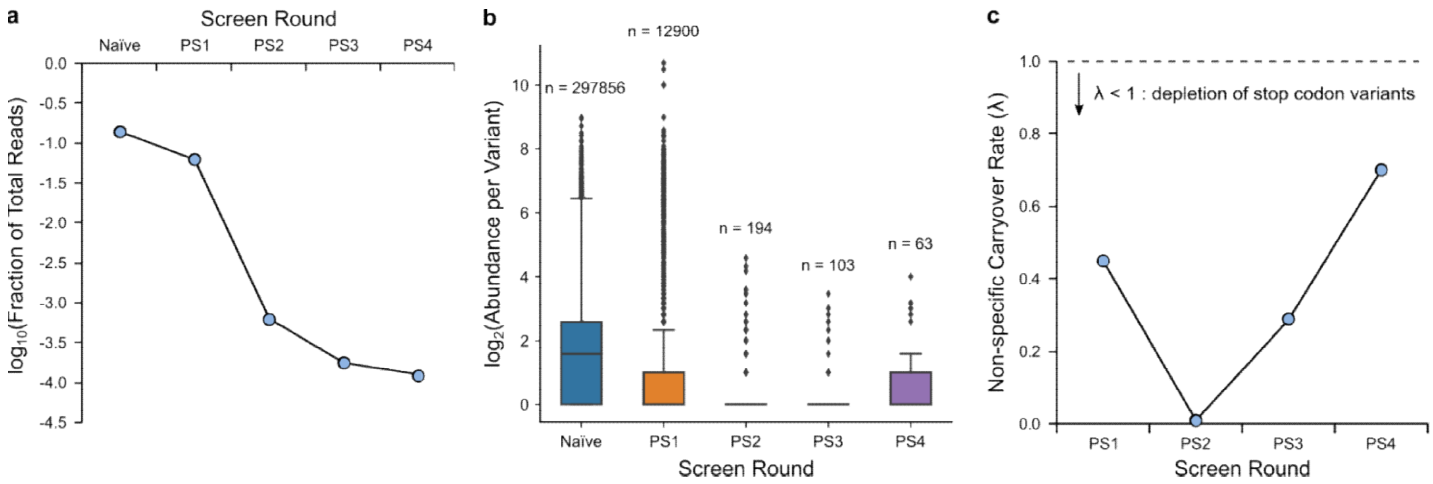

Supplementary Figure 8: A_2aR variants containing at least 1 stop codon are rapidly depleted from the library. (a) The fraction of reads containing at least 1 stop codon decreases by approximately 3 orders of magnitude after 4 rounds of sorting. (b) The distribution of the $\log_2$(count) of unique variants containing at least 1 stop codon is displayed as a box plot for each population. As expected, both the mean abundance of each variant and the number (n) of unique inactive variants decrease rapidly during the screen. (c) The depletion of stop codon variants can be used to estimate the non-specific carryover rate (λ), where a value less than 1 indicates a decrease in the fraction of stop codon-containing reads compared to the previous round. The estimated non-specific carryover rate falls between 0.01 – 0.7 for each round of sorting. In each box plot, the box represents the 25th to the 75th percentile of each population. The middle line, if present, represents the median of each population. Whiskers in the box plot represent 1.5 times the interquartile range (IQR). Diamonds represent individual data points falling outside 1.5xIQR and span the minimum and maximum values.

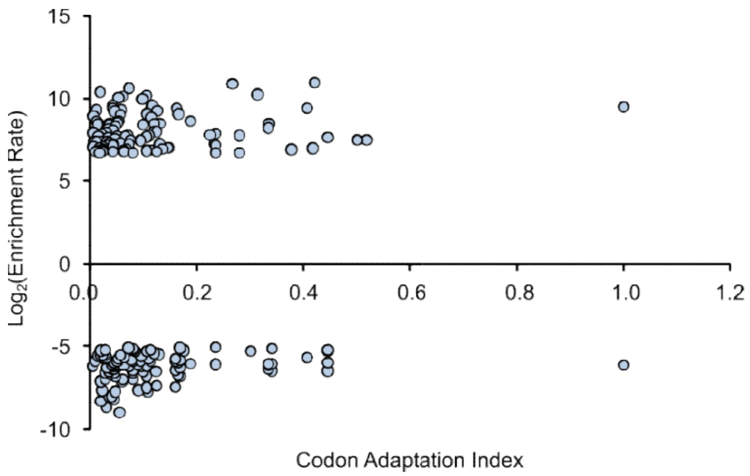

Supplementary Figure 9: The 100 most highly enriched and depleted A_2aR variants do not correlate with codon adaptation index indicating codon bias does not influence enrichment. The log2(enrichment rate) for each of the 100 most highly enriched and depleted A_2aR variants shows no correlation with the variant's codon adaptation index (CAI). CAI is a measure of the similarity of codon usage compared to a user-defined reference, where 0 indicates dissimilarity and 1 indicates complete similarity. Here, the codon usage reference is the one given in Sharp *et al.*[9], which was composed based on the codon usage in *Saccharomyces cerevisiae* genes that are subject to selection via translational efficiency.

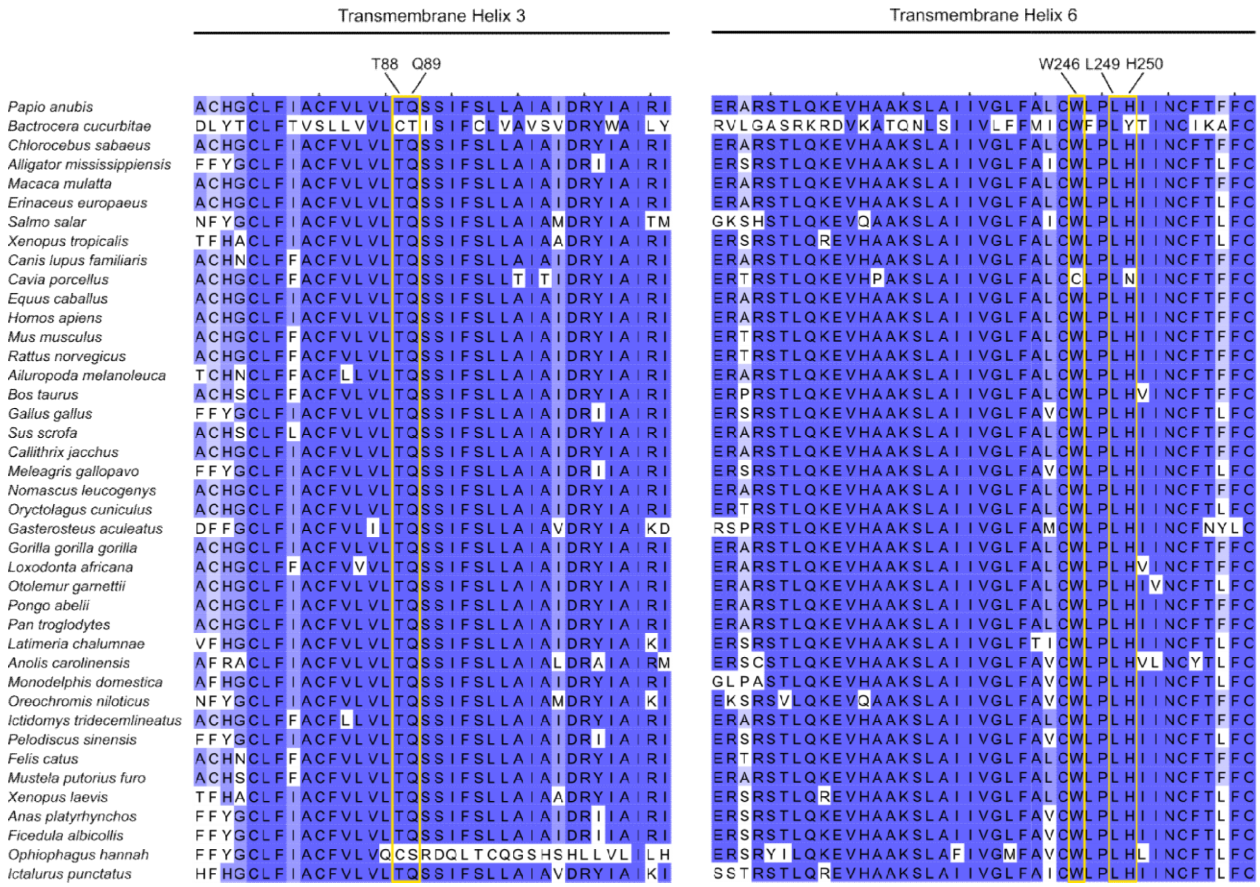

Supplementary Figure 10: Amino acid sequence alignment of A_2aR transmembrane helices 3 and 6 reveal high conservation of mutated residues among orthologs. The positions mutated in the A_2aR library (T88$^{3.36}$, Q89$^{3.37}$, W246$^{6.48}$, L249$^{6.51}$, and H250$^{6.52}$) are highly conserved across A_2aR orthologs suggesting important roles in receptor function. Ballesteros-Weinstein notation is used in superscript to denote the relative position of a residue with respect to the most evolutionarily conserved residue in a transmembrane helix. For example, the most conserved residue in helix 3 is 3.50. Sequence alignments were generated using jalview with sequences obtained through the GPCRdb.

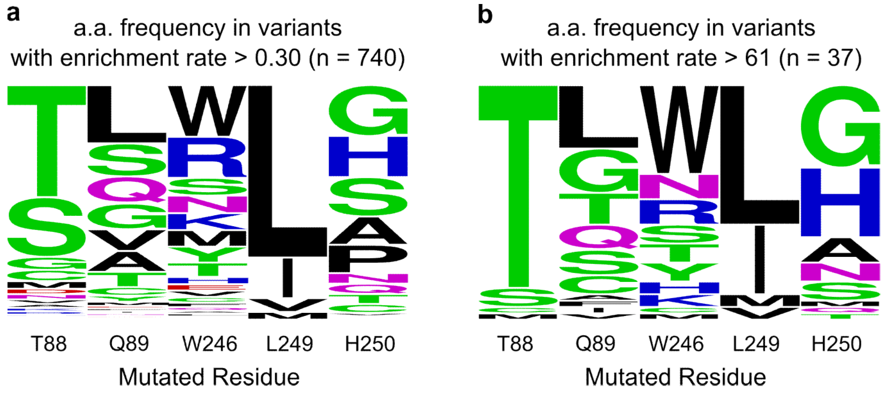

Supplementary Figure 11: Frequency logo plots for enriched PS4 variants reveal varying tolerance to substitutions among mutated sites. The amino acid (a.a.) frequency at each mutated position is plotted for variants in PS4 where the vertical scaling of each a.a. reflects its relative frequency at a specific position. The a.a. color reflects a residue's chemical properties where polar residues (G, S, T, Y, C, Q, and N) are green, basic residues (K, R, and H) are blue, acidic residues (D and E) are red, and hydrophobic residues (A, V, L, I, P, W, F, and M) are black. (a) Variants with PS4/naïve enrichment rates greater than 0.30, equal to the wildtype receptor rate, strongly favor T/S at T88 and L at L249. In contrast, sites Q89, W246, and H250 are permissive and sample a range of residues with varying chemical properties. Variants with PS4/naïve enrichment rates greater than 61, equivalent to the 95th percentile, exhibit similar preferences for sites Q89, W246, and L249. However, variants within this population more strongly prefer the wildtype residue at T88 and G/H at H250.

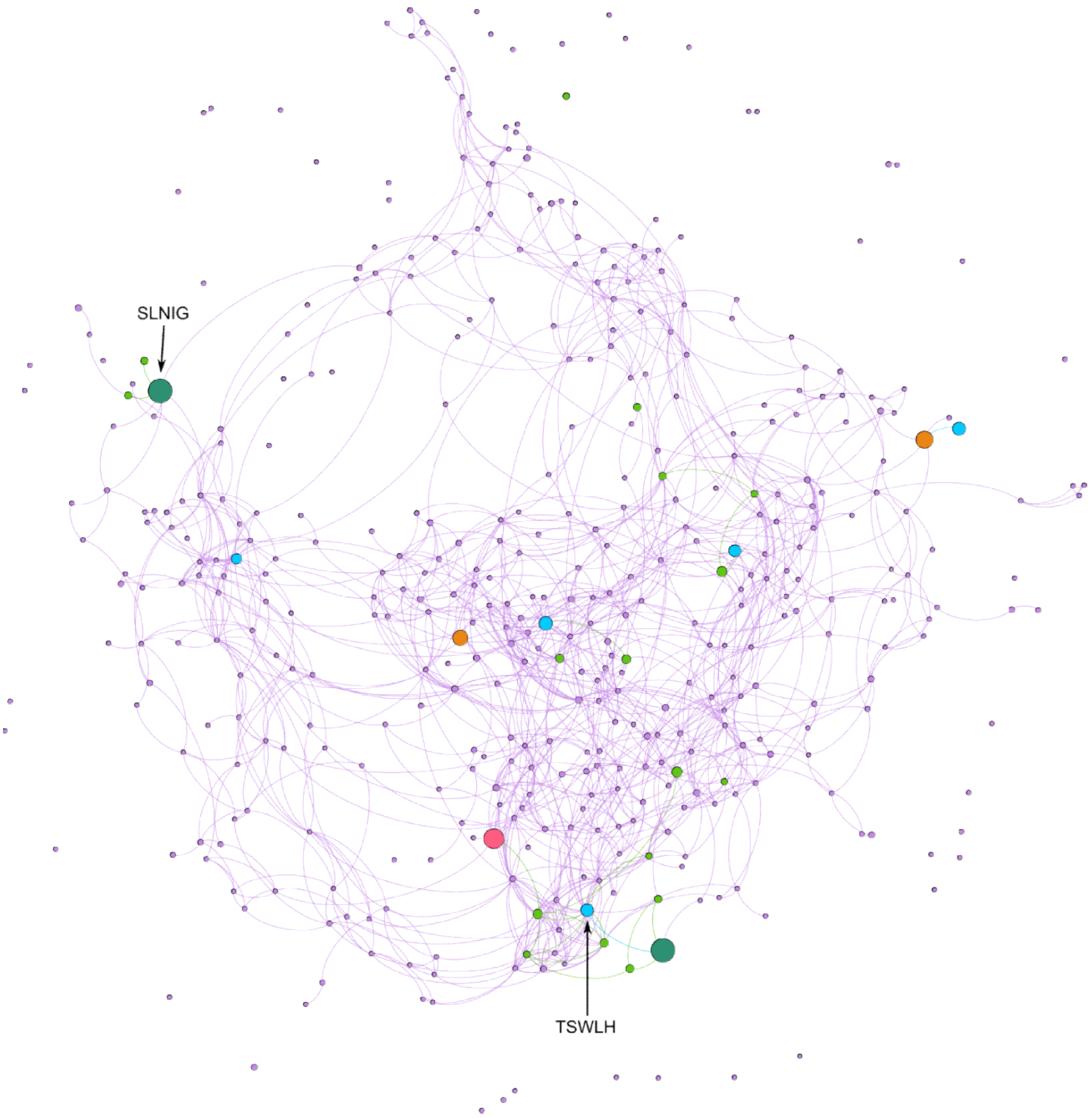

Supplementary Figure 12: Force-directed graph of PS4 variants with enrichment rates ≥ 1 reveals significant connectivity between modestly-enriched variants. Identical to Figure 3a, each node represents a unique variant, the node's radius scales with the variant's enrichment rate, each edge represents a difference in 1 amino acid, and the color of each edge is that of the variant with lower enrichment. Nodes are attracted to other nodes with which it shares an edge and repulsed from disconnected nodes. As a result, highly-interconnected nodes are clustered. These clusters largely consist of modestly-enriched (purple) nodes representing sequences more likely to be sampled through Darwinian evolution. Larger, isolated nodes can be imagined as taller peaks in a sequence-function landscape, which reflects epistatic interactions between residues of those variants. This epistasis contributes to the rarity of accessing isolated nodes such as SLNIG in favor of more highly-connected nodes such as TSWLH.

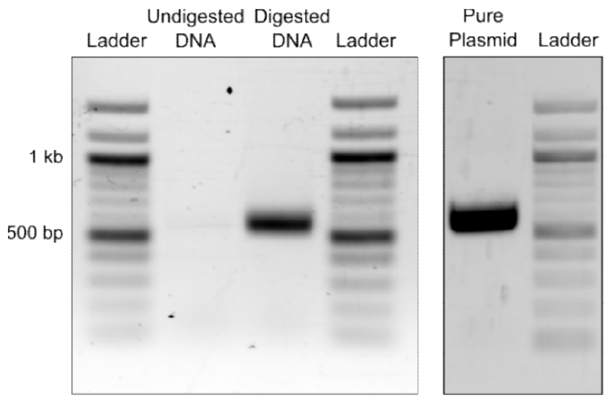

Supplementary Figure 13: Digestion of yeast DNA extract improves amplification of the targeted sequencing region. Preparation for next-generation sequencing was initially hindered by poor amplification of the target region within A_2aR from yeast total DNA extract (Undigested DNA). Digestion of DNA extract with *EcoRI* and *HindIII* prior to PCR significantly improves the yield of amplicon (Digested DNA) with the expected size (Pure Plasmid). In the PCRs using extracted DNA as template, 2 ng/µL DNA was used while 1 ng/µL plasmid (pYC A_2aR) was used in the PCR with pure plasmid. The total DNA extract and pure plasmid were run on separate gels, which were processed in parallel. Each gel contains the same mass of Ladder to facilitate comparison of DNA quantity across gels. These gel images represent the results of a single experiment. Source data are provided as a Source Data file.

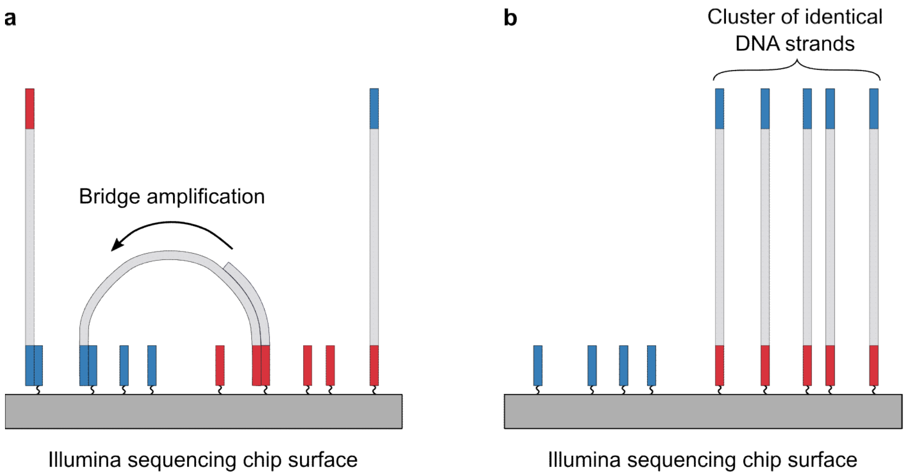

Supplementary Figure 14: Illustration of DNA strand attachment and bridge amplification on Illumina sequencing chip surface. (a) Illumina sequencing platforms feature two unique DNA adapters, which complement flanking sequences appended to a DNA strand (depicted in red and blue). Following interaction of complementary sequences in the strand and on the surface, the distal end of the bound strand approaches the surface facilitating binding to the second adapter. This second interaction enables bridge amplification, wherein the strand is used as a template for DNA synthesis from the second adapter. (b) Repeated cycles of bridge amplification produce clusters of identical DNA template strands covalently linked to the sequencing chip surface.

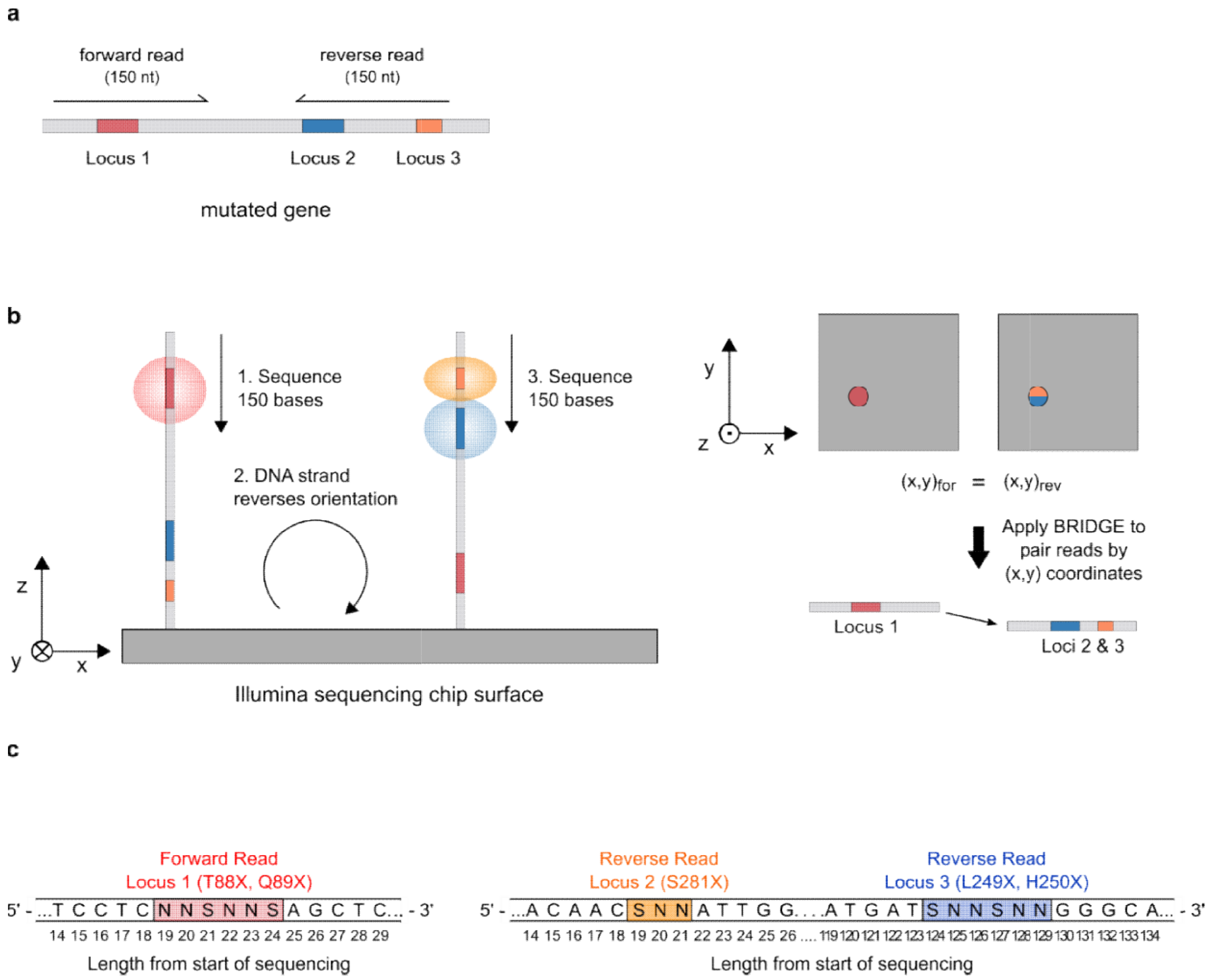

Supplementary Figure 15: Application of BRIDGE to a protein library containing three distal loci. (a) BRIDGE can be applied to match distal mutations placed in multiple loci as long as each locus falls within either the forward or reverse read. As an example, we describe use of BRIDGE to analyze an A_2aR library containing mutations at positions T88$^{3.36}$ and Q89$^{3.37}$ (locus 1), L249$^{6.51}$ and H250$^{6.52}$ (locus 2), and S281$^{7.46}$ (locus 3). (b) During paired-end sequencing, the reverse read (step 3) captures the nucleotide sequence at both locus 2 and 3. The forward and reverse reads will share an (x,y) coordinate on the sequencing chip surface enabling *in silico* matching of non-overlapping reads. (c) Practical considerations (e.g. sequencing accuracy as a function of read length) should be made for each library. The library design discussed in this example would place locus 3 near the 3' end of the reverse read, where sequencing errors are more abundant.

Supplementary Table 1: Intermolecular contacts made between A_2aR residues and ligands in various crystal structures

Residue	Ballesteros-Weinstein Notation	Transmembrane Domain	Total Number of Atomic Contacts
ALA 63	2.61	TM 2	3
ILE 66	2.64		3
SER 67	2.65		10
ALA 81	3.29	TM 3	4
VAL 84	3.32		8
LEU 85	3.33		7
THR 88	3.36		5
ALA 89[a]	3.37		2
PHE 168	N/A	N/A (ECL 2)	119
GLU 169	N/A		7
MET 177	5.38	TM 5	23
ASN 181	5.42		2
CYS 185	5.46		2
TRP 246	6.48	TM 6	6
LEU 249	6.51		37
HIS 250	6.52		14
ASN 253	6.55		26
LEU 267	7.32	TM 7	1
MET 270	7.35		5
TYR 271	7.36		5
ILE 274	7.39		26
ALA 277[b]	7.42		2
SER 277	7.42		4
HIS 278	7.43		15

[a]Q89A, [b]S277A

PDB IDs: 2YDO, 2YDV, 3PWH, 3REY, 3RFM, 3UZA, 3UZC

Supplementary Table 2: Changes in NECA binding affinities and enrichment rates for internal library controls

Variant	Fold change in NECA binding affinity relative to wildtype A_2aR	High Stringency Enrichment
WT	N/A	0.30
T88A	0.012[a], 0.005[b]	0.49
T88D[c]	NB[g]	ND[h]
T88E[c]	NB	0.0033
T88R[a]	0.007	0.021
T88S[a]	0.025	0.14
Q89A[a]	15	149
Q89D[c]	14	0.73
W246A[d]	0.042	ND
H250N[e]	4	4.1
H250F[f]	0.47	ND
H250Y[f]	0.45	ND

[a]Jianget al.[5], [b]Bertheleme et al.[10], [c]Kim et al.[11], [d]Massink et al.[12], [e]Jiang et al.[13], [f]Kim et al.[4], [g]NB, no specific binding detected, [h]ND, no variants detected in PS4

Supplementary Table 3: Average quality (Q) scores and predicted error rates of variable bases in the forward and reverse sequencing reads

Library	Forward Read Mutated Bases Q Score	Forward Read Predicted Error Rate	Reverse Read Mutated Bases Q Score	Reverse Read Predicted Error Rate
Naïve	34.7	0.034%	34.9	0.032%
PS1	35.0	0.031%	34.9	0.032%
PS2	35.0	0.032%	34.7	0.034%
PS3	35.0	0.032%	35.0	0.032%
PS4	35.2	0.030%	35.1	0.031%

Supplementary Table 4: Average quality (Q) scores, predicted error rates, and observed error rates of the bases immediately upstream and downstream of each variable region

Library	Forward Read Flanking Bases Q Score	Forward Read Predicted % Error	Forward Read Observed % Error	Reverse Read Flanking Bases Q Score	Reverse Read Predicted % Error	Reverse Read Observed % Error
Naïve	35.3	0.029%	0.029%	35.3	0.029%	0.048%
PS1	35.3	0.029%	0.025%	34.9	0.032%	0.038%
PS2	35.0	0.032%	0.029%	34.7	0.034%	0.035%
PS3	35.4	0.029%	0.024%	35.0	0.032%	0.033%
PS4	35.2	0.030%	0.028%	35.3	0.030%	0.021%

Supplementary Table 5: Top 25 variants ranked by Enrich2 score

Variant	Enrich2 Score
TGRIA	5.76
TGRLG	5.36
TGRIS	5.32
TGHLA	5.06
TTTIG	4.85
TCYLH	4.84
TGNIG	4.74
TSWIH	4.72
TGRLC	4.70
TTYLH	4.69
TQWIN[a]	4.65
DVKMQ	4.62
TLHMG	4.60
TQNIG[b]	4.55
DVHIP	4.52
TGHIA	4.51
TGWIH	4.50
TGRLS	4.46
TGRLA	4.40
SLTLQ	4.39
TGKLA	4.30
TANIG	4.30
TLTLN	4.27
TTNLG	4.26
TLVIN	4.24

[a]proximal double residue mutant, [b]proximal triple residue mutant

Supplementary Table 6: Primer sequences (5' to 3')

Primer #	Primer Name	Sequence (5' to 3')
1	HindIII prepro-A2a for	CTATAAGCTTGGATCATGAAGGTTTTGATTGT
2	A2aXhoIrev	TGACCTCGAGCCGTACAGTAATAGATACCCATG
3	A2a Q89A for	CGTCCTGGTCCTCACGGCTAGCTCCATCTTCAG
4	A2a Q89A rev	CTGAAGATGGAGCTAGCCGTGAGGACCAGGACG
5	A2a Q89S for	CGTCCTGGTCCTCACGTCCAGCTCCATCTTCAG
6	A2a Q89S rev	CTGAAGATGGAGCTGGACGTGAGGACCAGGACG
7	A2a T88C Q89V for	CGTCCTGGTCCTCTGCGTGAGCTCCATCTTCAG
8	A2a T88C Q89V rev	CTGAAGATGGAGCTCACGCAGAGGACCAGGACG
9	A2a W246R L249I H250C for	CTTTGCCCTCTGCCGCCTGCCCATCTGCATCATCAACTGC
10	A2a W246R L249I H250C rev	GCAGTTGATGATGCAGATGGGCAGGCGGCAGAGGGCAAAG
11	A2a Q89G for	CGTCCTGGTCCTCACGGGCAGCTCCATCTTCAG
12	A2a Q89G rev	CTGAAGATGGAGCTGCCCGTGAGGACCAGGACG
13	A2a W246R L249I H250A for	CTTTGCCCTCTGCAGGCTGCCCATCGCCATCATCAACTGC
14	A2a W246R L249I H250A rev	GCAGTTGATGATGGCGATGGGCAGCCTGCAGAGGGCAAAG
15	A2a T88S Q89L for	CGTCCTGGTCCTCAGCCTCAGCTCCATCTTCAG
16	A2a T88S Q89L rev	CTGAAGATGGAGCTGAGGCTGAGGACCAGGACG
17	A2a W246N L249I H250G for	CTTTGCCCTCTGCAACCTGCCCATCGGCATCATCAACTGC
18	A2a W246N L249I H250G rev	GCAGTTGATGATGCCGATGGGCAGGTTGCAGAGGGCAAAG
19	A2a T88X Q89X for	CGTCCTGGTCCTCNNSNNSAGCTCCATCTTCAG
20	A2a T88X Q89X rev	CTGAAGATGGAGCTSNNSNNGAGGACCAGGACG
21	A2a W246X L249X H250X for	CTTTGCCCTCTGCNNSCTGCCCNNSNNSATCATCAACTGC
22	A2a W246X L249X H250X rev	GCAGTTGATGATSNNSNNGGGCAGSNNGCAGAGGGCAAAG
23	NGS A2a for	TCGTCGGCAGCGTCAGATGTGTATAAGAGACAGTGCTTCGTCCT GGTCCTC
24	NGS A2a for 1	TCGTCGGCAGCGTCAGATGTGTATAAGAGACAGCTGCTTCGTC CTGGTCCTC
25	NGS A2a for 2	TCGTCGGCAGCGTCAGATGTGTATAAGAGACAGACTGCTTCGT CCTGGTCCTC
26	NGS A2a for 3	TCGTCGGCAGCGTCAGATGTGTATAAGAGACAGGAATGCTTCG TCCTGGTCCTC
27	NGS A2a rev	GTCTCGTGGGCTCGGAGATGTGTATAAGAGACAGGCAGAAGA AAGTGAAGCAGTTG
28	NGS A2a rev 1	GTCTCGTGGGCTCGGAGATGTGTATAAGAGACAGAGCAGAAG AAAGTGAAGCAGTTG
29	NGS A2a rev 2	GTCTCGTGGGCTCGGAGATGTGTATAAGAGACAGCTGCAGAAG AAAGTGAAGCAGTTG
30	NGS A2a rev 3	GTCTCGTGGGCTCGGAGATGTGTATAAGAGACAGTATGCAGAA GAAAGTGAAGCAGTTG

Supplementary References

1. Gupta, K. & Varadarajan, R. Insights into protein structure, stability and function from saturation mutagenesis. *Curr. Opin. Struct. Biol.***50**, 117–125 (2018).

2. Metzker, M. L. Sequencing technologies - the next generation. *Nat. Rev. Genet.***11**, 31–46 (2010).

3. Kircher, M. & Kelso, J. High-throughput DNA sequencing - Concepts and limitations. *BioEssays***32**, 524–536 (2010).

4. Kim, J., Wess, J., Michiel van Rhee, A., Schoneberg, T. & Jacobson, K. A. Site-directed Mutagenesis Identifies Residues Involved in Ligand Recognition in the Human A2a Adenosine Receptor. *J. Biol. Chem.***270**, 13987–13997 (1995).

5. Jiang, Q. *et al.* Hydrophilic side chains in the third and seventh transmembrane helical domains of human A2a adenosine receptors are required for ligand recognition. *Mol. Pharmacol.***50**, 512–521 (1996).

6. Head, S. R. *et al.* Library construction for next-generation sequencing: Overviews and challenges. *Biotechniques***56**, 61–77 (2014).

7. Mardis, E. R. Next-Generation Sequencing Platforms. *Annu. Rev. Anal. Chem.***6**, 287–303 (2013).

8. Lebon, G. *et al.* Agonist-bound adenosine A2A receptor structures reveal common features of GPCR activation. *Nature***474**, 521–525 (2011).

9. Sharp, P. M. & Li, W. H. The codon Adaptation Index--a measure of directional synonymous codon usage bias, and its potential applications. *Nucleic Acids Res.***15**, 1281–95 (1987).

10. Bertheleme, N., Singh, S., Dowell, S. J., Hubbard, J. & Byrne, B. Loss of constitutive activity is correlated with increased thermostability of the human adenosine A2A receptor. *Br. J. Pharmacol.***169**, 988–998 (2013).

11. Kim, S. *et al.* Modeling the adenosine receptors: comparison of the binding domains of A2A agonists and antagonists. *J. Med. Chem.***46**, 4847–59 (2003).

12. Massink, A. *et al.* Sodium Ion Binding Pocket Mutations and Adenosine A2A Receptor Function. *Mol. Pharmacol.***87**, 305–313 (2015).

13. Jiang, Q., Lee, B. X., Glashofer, M., Van Rhee, a. M. & Jacobson, K. A. Mutagenesis reveals structure-activity parallels between human A(2A) adenosine receptors and biogenic amine G protein-coupled receptors. *J. Med. Chem.***40**, 2588–2595 (1997).
