# Supplementary material for: Bridging non-overlapping reads illuminates high-order epistasis between distal protein sites in a GPCR
Source: Nat Commun. 2020 Feb 4;11:690. doi: 10.1038/s41467-020-14495-7 (PMC7000732; doi:10.1038/s41467-020-14495-7)
Supplement: Supplementary file 2 — Reporting Summary [file 41467_2020_14495_MOESM2_ESM.pdf]

nature research

Corresponding author(s): Michelle O'Malley

Last updated by author(s): Dec 30, 2019

Reporting Summary

Nature Research wishes to improve the reproducibility of the work that we publish. This form provides structure for consistency and transparency in reporting. For further information on Nature Research policies, see Authors & Referees and the Editorial Policy Checklist .

Statistics

For all statistical analyses, confirm that the following items are present in the figure legend, table legend, main text, or Methods section.

n/a	Confirmed	
☐	☒	The exact sample size (*n*) for each experimental group/condition, given as a discrete number and unit of measurement
☐	☒	A statement on whether measurements were taken from distinct samples or whether the same sample was measured repeatedly
☐	☒	The statistical test(s) used AND whether they are one- or two-sided<br>*Only common tests should be described solely by name; describe more complex techniques in the Methods section.*
☒	☐	A description of all covariates tested
☒	☐	A description of any assumptions or corrections, such as tests of normality and adjustment for multiple comparisons
☒	☐	A full description of the statistical parameters including central tendency (e.g. means) or other basic estimates (e.g. regression coefficient) AND variation (e.g. standard deviation) or associated estimates of uncertainty (e.g. confidence intervals)
☒	☐	For null hypothesis testing, the test statistic (e.g. *F*, *t*, *r*) with confidence intervals, effect sizes, degrees of freedom and *P* value noted<br>*Give P values as exact values whenever suitable.*
☒	☐	For Bayesian analysis, information on the choice of priors and Markov chain Monte Carlo settings
☒	☐	For hierarchical and complex designs, identification of the appropriate level for tests and full reporting of outcomes
☒	☐	Estimates of effect sizes (e.g. Cohen's *d*, Pearson's *r*), indicating how they were calculated

Our web collection on statistics for biologists contains articles on many of the points above.

Software and code

Policy information about availability of computer code

Data collection	No software was used for data collection.
Data analysis	Custom code (available in the submitted code folder) was used to process and analyze raw .fastq NGS sequencing files. The custom code requires installation of biopython (1.73 or later) and CAI (v1.0.3 or later). Enrich2 (v1.2.0) was used to analyze the fitness of variants in addition to our method/code (PS4/naive enrichment rate).

For manuscripts utilizing custom algorithms or software that are central to the research but not yet described in published literature, software must be made available to editors/reviewers. We strongly encourage code deposition in a community repository (e.g. GitHub). See the Nature Research guidelines for submitting code & software for further information.

Data

Policy information about availability of data

All manuscripts must include a data availability statement. This statement should provide the following information, where applicable:

- Accession codes, unique identifiers, or web links for publicly available datasets
- A list of figures that have associated raw data
- A description of any restrictions on data availability

Data Availability
Raw sequencing data generated in this study have been deposited in the NCBI Sequence Read Archive under accession number PRJNA545342.
Code Availability
Python code generated in this work is available frrom https://github.com/j-yoo/BRIDGE.

Field-specific reporting

Please select the one below that is the best fit for your research. If you are not sure, read the appropriate sections before making your selection.

☒ Life sciences ☐ Behavioural & social sciences ☐ Ecological, evolutionary & environmental sciences

For a reference copy of the document with all sections, see nature.com/documents/nr-reporting-summary-flat.pdf

Life sciences study design

All studies must disclose on these points even when the disclosure is negative.

Sample size	A sample size of 3 biological yeast (S. cerevisiae) replicates was used for radioligand binding and flow cytometric analysis with the exception of the data presented in Supplementary Figure 3. For flow cytometric analyses, ~100,000 events were collected for each sample. This sample size is in accordance with literature standards.
Data exclusions	No data were excluded from this analysis.
Replication	3 independent, biological replicates were included for the radioligand binding and flow cyotmetric analyeses. All replicates demonstrated high degrees of reproducibility in each experiment.
Randomization	Randomization was not relevant to this study as samples were grouped for experiments or analyses. Controls and experimental samples were not sourced from the same group (i.e. strain).
Blinding	Blinding was not relevant to this study as samples were not grouped for any experiments or analyses.

Reporting for specific materials, systems and methods

We require information from authors about some types of materials, experimental systems and methods used in many studies. Here, indicate whether each material, system or method listed is relevant to your study. If you are not sure if a list item applies to your research, read the appropriate section before selecting a response.

Materials & experimental systems

n/a	Involved in the study	
☒	☐	Antibodies
☒	☐	Eukaryotic cell lines
☒	☐	Palaeontology
☒	☐	Animals and other organisms
☒	☐	Human research participants
☒	☐	Clinical data

Methods

n/a	Involved in the study	
☒	☐	ChIP-seq
☐	☒	Flow cytometry
☒	☐	MRI-based neuroimaging

Flow Cytometry

Plots

Confirm that:

☒ The axis labels state the marker and fluorochrome used (e.g. CD4-FITC).

☒ The axis scales are clearly visible. Include numbers along axes only for bottom left plot of group (a 'group' is an analysis of identical markers).

☒ All plots are contour plots with outliers or pseudocolor plots.

☒ A numerical value for number of cells or percentage (with statistics) is provided.

Methodology

Sample preparation	Yeast cells producing A2aR were diluted to an OD600of 1.0 in 1 mL binding buffer (50 mM Tris-HCl, 10 mM MgCl2, pH = 7.5) and incubated with FITC-APEC (NIMH, Bethesda, MD) with gentle mixing while protected from light at room temperature for 1 hour. Cells were then placed on ice for 30 minutes prior to resuspension in 1 mL ice-cold 1X phosphate buffered saline (PBS, pH = 7.5) and immediate analysis using FACS.
Instrument	BD FACSAria I flow cytometer
Software	FlowJo V10.0.7r2

Cell population abundance	In the first round of screening ~50,000 cells were collected, while ~100,000 cells were collected in each subsequent round of screening. Cells were sorted at an event rate ~5000 event/s.
Gating strategy	During the first 2 rounds of sorting, yeast harboring the A2aR library were sorted to collect all cells with green fluorescence intensities greater than ~99.5% of the negative control. Beginning with the third round of screening, the post sort (PS) 2 population was sorted to collect all cells with fluorescence intensities above background (low-stringency) or 5% of the population with the greatest fluorescence intensities (high-stringency). In the fourth round of screening, the PS3 populations isolated using low- or high-stringency gating were sorted using the same strategy as in the previous round. Analysis of the naive library indicated 0.56% of the population fell within the sort gate used during the first 2 rounds of screening (Supplementary Figure S5). Approximately 13% of a positive control (cells producing wildtype A2aR) fell within the sort gate. Approximately 0.5% of a negative control (cells not producing A2aR) fell within the sort gate.

☒ Tick this box to confirm that a figure exemplifying the gating strategy is provided in the Supplementary Information.
